# The interplay between residency program culture and feedback culture: a cross-sectional study exploring perceptions of residents at three institutions

**DOI:** 10.1080/10872981.2019.1611296

**Published:** 2019-04-30

**Authors:** Robert Bing-You, Subha Ramani, Saradha Ramesh, Victoria Hayes, Kalli Varaklis, Denham Ward, Maria Blanco

**Affiliations:** a Department of Medical Education, Maine Medical Center, Portland, ME, USA; b Department of Medicine, Brigham and Women’s Hospital, Boston, MA, USA; c Office of Educational Affairs, Tufts University School of Medicine, Boston, MA, USA

**Keywords:** Feedback, culture, organizational, residency, hierarchy

## Abstract

**Background**: Giving and receiving feedback that changes performance is influenced significantly by the clinical learning environment. This environment is multi-dimensional but includes both organizational and feedback specific dimensions.

**Objective**: The objectives of this research were to investigate the relationship between residents’ perceptions of residency program culture and feedback culture; and whether there were differences in resident perceptions of their programs’ and feedback cultures based on their disciplines and institution. We hypothesized that residents preferred certain program culture types and that certain aspects of a residency program’s culture were related to the feedback culture.

**Design**: Residents from six specialties at three institutions voluntarily completed two validated survey instruments (Organizational Culture Assessment Instrument [OCAI] and Feedback in Medical Education [FEEDME]-Culture survey) to assess the residency program and feedback cultures, respectively. Descriptive statistics were calculated and non-parametric tests were used to analyze the data.

**Results**: The overall response rate was 37.9% (116/306 residents). ‘Clan’ culture was both the current and preferred culture by 49.3% and 56.8%, respectively, of the residents overall. There were differences across programs with more current ‘clan’ culture in pediatrics than in surgery (P = 0.01). Multiple regression analysis showed the Hierarchy Now culture type was significantly related to the feedback culture mean score (p = <.01). For every one unit increase in the Hierarchy Now culture type, the FEEDME-Culture mean score decreases by 0.023 units.

**Conclusions**: The findings of this study add to the literature by describing residents’ preferences of their residency program’s culture, and providing insights into the interplay between the residency program and feedback cultures.

## Introduction

Recent literature on feedback in medical education strongly emphasizes bidirectional dialogue and exchange of information [1], described as an ‘educational alliance’ between the learner and the teacher, and recommends coaching for professional growth rather than ‘giving’ feedback [2–4]. While the newer conceptual models of this complex, interpersonal interaction [5] sound promising, discussion on promoting feedback seeking and acceptance by learners cannot be devoid of the context (i.e., learning environment) within which the feedback occurs [6,7]. As suggested by the popularized phrase ‘culture eats strategy for breakfast,’ [8] efforts to enhance the exchange of feedback within an educational alliance could be drastically undermined if the culture of an educational program does not support the newer approaches to feedback. In order to enhance the quality and impact of feedback, understanding the residency program culture (i.e., defined as the underlying assumptions and values that drive the behaviors of an organization) and addressing sociocultural barriers are paramount.

Two perspectives of culture might impact the feedback process between faculty and medical students and residents: the learning culture and the residency program culture. Learning culture is described as the attitudes, values and practices that impact how a profession designs education for its learners [9]. It can affect the feedback-giving behavior of medical teachers [10] the feedback seeking behavior of learners, the credibility of feedback, and the process and outcome of feedback [11]. Watling et al. [12,13] explored the impact of the learning culture on feedback in three different professional contexts (e.g., medicine, music, professional sports) and found significant differences in feedback seeking, acceptance of constructive feedback and direct observation of performance. This research suggests professions may have a unique and dominant learning culture influencing the feedback process.

The organizational culture (or what we have called ‘residency program culture’) drives the behaviors of an organization that can either facilitate or inhibit educational efforts [14]. For example, it has been reported that medical schools with a culture that promotes flexible policies and interdisciplinary teamwork implement curricular reform more successfully than medical schools with authoritative cultures [15]. We believe that a residency program exists within a departmental structure that could have a specific or dominant culture. In a qualitative study, Ramani et al. [16] reported that cultural factors dominated internal medicine residents’ perceptions of the quality and impact of feedback; interestingly, a departmental ‘culture of niceness’ was viewed by residents as a barrier rather than as a promoter of meaningful feedback. Sargeant et al. [4] examined the implementation of the R2C2 model (relationship building; exploring reactions to feedback; exploring understanding of feedback content; and coaching for performance change) in internal medicine and pediatrics programs as a potential strategy to shift towards a culture with more positive feedback.

The Organizational Culture Assessment Instrument (OCAI) is a well-known framework for assessing the culture of a work environment [17], although the use of the OCAI in graduate medical education is novel. The OCAI differentiates culture along two dimensions: one concentrated on external versus internal orientation, and the other concentrated on discretion and flexibility versus stability and control. These two dimensions identify how individuals view the culture of their work environments within one of four distinct frames: Clan, Hierarchy, Adhocracy, and Market. A Clan culture reflects a more sociable working environment, and individuals view it as a ‘family.’ Hierarchy cultures focus on structure and formality, with an emphasis on organizations functioning efficiently. The Adhocracy culture reflects a climate where risk is accepted in an innovative, energetic, and creative environment. Market cultures are results-oriented, and individuals complete goals in a competitive manner. Efforts to measure culture have mainly occurred in other professional fields rather than in medical education [18]. However, Rougas et al. [19] developed a modified OCAI tool, and collected validity evidence of the tool implementation within an emergency medicine residency program.

We define feedback as information given to learners for the purpose of improving their performance, and we concur with the theoretical constructs of the feedback exchange as an educational alliance [2]. That said, due to the lack of validated instruments to assess learner’s perception of the feedback they receive, we developed such instruments and collected preliminary validity evidence in a previous study [20]. Our previous work determined that learners differentiate between the feedback received from the milieu or environment of a rotation (i.e., what we have termed ‘culture’) versus the feedback received from an individual provider.

There is a lack of studies that examine the culture in residency programs, especially as it pertains to the influence of this residency program culture on the exchange of feedback, the perception of its credibility, and the impact of feedback on behavior change. Since a greater understanding of the residency program and feedback cultures is necessary to enhance the quality and impact of the feedback process, our research questions were: 1) What is the relationship between residents’ perceptions of their residency program’s culture and feedback culture?; and 2) Are there differences in residents’ perceptions of their programs’ and feedback cultures amongst residents from different disciplines and different institutions? We hypothesized that residents preferred certain program culture types and that certain aspects of a residency program’s culture were related to the feedback that occurred in a learning culture.

## Methods

### Setting and participants

Maine Medical Center (MMC) is an independent, urban, academic medical center with approximately 250 residents and fellows across 25 training programs. A convenience sampling approach was used to recruit residents from the Departments of Internal Medicine, Family Medicine, OB/GYN, Pediatrics, Psychiatry and Surgery. Internal Medicine residents from Boston Institution A (BIA), which is a large urban academic medical center and a teaching affiliate of Harvard Medical School, were also recruited as well as Pediatric residents from Boston Institution B (BIB). A total of 306 residents from the above residency programs at the three institutions were invited to participate in the study. Residency programs in the U.S. are completed typically after four years of undergraduate schooling (i.e., college) and four years of medical school training. Residency programs can vary from three to five years in length depending on the specialty, with residents commonly spending the majority of their training at one institution.

In May and June of 2017, the 306 residents noted above were asked to voluntarily complete the anonymous, electronic survey instruments either via an email invitation, or at one of their regularly scheduled residency conferences. The residents were from all postgraduate levels. There were no incentives provided for participation. A reminder was sent to residents at Boston Institution B two weeks after the initial invitation; no reminders were sent to the MMC or Boston Institution A residents. The research project was determined to be exempt research by the MMC Institutional Review Board.

### Study design

We designed a cross-sectional survey study. Participants completed two survey instruments: the OCAI [17] and the Feedback in Medical Education (FEEDME)-Culture survey [20]. We considered this study design to be appropriate since our research questions were related to the beliefs, opinions, and attitudes of the intended participants.

### Survey instruments

We used the Qualtrics (Provo, UT) program to administer the surveys. This program allows participants to access the survey link on any desktop or mobile device. We sent emails to residents with a single anonymous electronic link. Residents were offered an option to complete the surveys at one sitting or to save and return to the surveys later. Residents completed the OCAI survey first and the FEEDME-Culture instrument afterwards.

The OCAI [17] was slightly modified by replacing the word ‘organization’ with the words ‘residency program’ (electronic supplementary material). The OCAI consists of two sets of six questions to measure six domains – Dominant Characteristics, Organizational Leadership, Management of Employees, Organization Glue, Strategic Emphases and Criteria for Success. Each question has four alternatives (A = Clan, B = Adhocracy, C = Market, and D = Hierarchy). The first set of questions is labeled as ‘Now’ that refers to the existing culture. We asked the residents to divide 100 points among the four alternatives and give higher points to the options that were similar to their residency program culture. The same set of questions was repeated and labeled as ‘Preferred.’ With the second set, we asked the residents to allot points based on how they would like their residency program to look five years from now. The scores were calculated by taking the average of all alternative responses in the Now and Preferred sets. The Cronbach’s alpha value for the OCAI is 0.86 [21].

The FEEDME-Culture instrument (electronic supplementary material) comprises 16 items each with a 1–5 Likert-type rating scale. This tool assesses learners’ perceptions of the feedback culture in their learning environment [20]. A FEEDME-Culture mean score is calculated from the average of all 16 items. Since the mean score is an aggregate score of 5-point Likert scale items, now it takes on the property of a continuous scale. Higher scores indicate a more positive feedback culture, where learners perceive that they are receiving effective, beneficial, and quality feedback about their performance. The Cronbach value of the FEEDME-Culture instrument is 0.94.

### Analysis

We used SPSS 24.0 version for quantitative data analysis. We calculated descriptive statistics (e.g., means, standard deviations) for the OCAI scores and FEEDME-Culture items. Since the normality test was significant for the FEEDME-Culture mean score, we employed non-parametric Mann-Whitney U tests to assess any differences in FEEDME-Culture mean scores between specialties from different institutions (i.e., Internal Medicine residents from MMC and BIA, and Pediatric residents from MMC and BIB).

Furthermore, we examined the correlation between OCAI Now and Preferred culture types and applied multiple regression method to analyze the relationship between the FEEDME-Culture mean score and the OCAI scores of the Now culture type. We also conducted one-way multivariate analysis of variance (MANOVA) tests to examine whether mean differences among specialty groups were likely to have occurred by chance [22]. A priori power analysis suggested that for conducting MANOVA analysis, a minimum sample size of 103 is required to have 80% power for detecting a minimum effect size at the traditional statistical significance level of 0.05.

## Results

A total of 116 residents (37.9%) completed the survey instruments. An additional 18 surveys were not included in the analysis due to incomplete responses. Three Medicine-Pediatrics residents completed the surveys and their responses were included with the Pediatrics group. Table 1 shows the mean, standard deviation, median, and range values for each of the FEEDME-Culture items for all residency programs. Table 2 shows the number of residents who responded by institution and program. The response rate by program ranged from 10% to 93.3 %. Of the 116 residents, the percentage of PGY1, PGY2, PGY3, PGY4, PGY5 and higher levels was 33%, 32%, 28%, 4%, and 3%, respectively.

**Table 1.** Mean and standard deviation of FEEDME-culture survey items (n = 116).

	Mean^a^	Std. Deviation	Median	Range
1. Indicate how often you received feedback in this rotation.	3.45	0.79	3.00	4
2. Before I was given the feedback, I was asked for a self-assessment of my performance I had performed.	3.16	0.96	3.00	4
3. The feedback that I received contained specific details about my performance.	3.47	0.82	3.00	4
4. The feedback prompted me to reflect on my performance.	3.76	0.83	4.00	4
5. The feedback included suggestions to help me improve.	3.49	0.76	3.00	4
6. When I was given feedback on how to improve, I felt the expectations were reasonable and feasible.	3.67	0.81	4.00	4
7. Time was set aside to give me feedback.	3.28	0.90	3.00	4
8. I received the feedback in time for me to act on it.	3.21	0.81	3.00	4
9. The feedback I received was based on direct observations of my performance.	3.82	0.90	4.00	4
10. The feedback helped me improve my performance.	3.50	0.75	3.00	4
11. The feedback that I received in this rotation helped me identify my strengths and weaknesses.	3.45	0.78	3.00	4
12. The learning environment of this rotation allowed me to try out the feedback that I received.	3.46	0.77	3.00	4
13. I understood the purpose of the feedback that I received during this rotation.	3.73	0.81	4.00	4
14. I received reinforcing (what I should keep doing) and corrective (what I need to work on) feedback.	3.49	0.87	4.00	4
15. There was follow-up on the feedback that I received in order to review my progress.	2.97	0.92	3.00	4
16. The amount of feedback I received met my learning needs.	3.32	0.85	3.00	4
Overall, how would you rate the quality of the feedback you received in this rotation?	3.53^b^	0.85	3.00	4
Feedback-Culture Mean Score	3.45	0.62	3.44	4

^a^Likert-type scale 1 = Never to 5 = Always for items #1 through #16. ^b^Likert-type scale 1 = Very Poor to 5 = Excellent.

**Table 2.** Descriptive statistics of FEEDME-culture and OCAI surveys for all residency programs.

			OCAI Culture type, Now (N) and Preferred (P). Mean % agreeing							
			Clan		Adhocracy		Market		Hierarchy	
	Nr	FEEDME Culture Mean score	N	P	N	P	N	P	N	P
All residents ^a^	116	3.45	49.3	56.8	16.4	19.6	11.7	7.8	22.5	16.0
Maine Medical Center (MMC)	87	3.45	51.0	57.2	15.7	19.2	9.6	7.4	23.7	16.5
Internal Medicine	19	3.25	53.9	61.7	16.6	19.1	7.5	6.3	21.6	12.9
Pediatrics	15	3.49	58.6	62.9	14	18.1	5.4	3.1	21.5	15.9
Psychiatry	14	3.94	51.2	68.5	17.9	15.7	8.5	3.3	22.4	13.8
OB/GYN	10	2.91	43.7	46.7	13.5	18.8	11.2	12.4	31.7	22.2
Family Medicine	16	3.62	54.8	58.1	15.2	21.3	9.3	5.9	20.7	14.7
Surgery	13	3.36	38.5	39.1	15.9	21.4	17.4	16	28.2	23.5
Boston Institution A Internal Medicine (BIA)	14	3.49	57.9	64.5	17.9	17.2	8.5	6.2	15.6	12
Boston Institution B Pediatrics (BIB)	15	3.44	31.7	47.1	19.4	24.1	26.5	11.8	22.2	16.9

^a^ – excludes 18 unfinished surveys. Nr – number. FEEDME-Culture results (means) based on Likert-type scale 1 = Never to 5 = Always.

### Residents’ perceptions of their programs’ organizational culture (OCAI survey)

Table 2 shows MMC, BIA, and BIB scores of both Now and Preferred culture types (A = Clan, B = Adhocracy, C = Market, and D = Hierarchy) for each residency program, and the FEEDME-Culture mean scores.

The reliability analysis findings indicated that the Cronbach’s alpha values of the collected data were reliable as the alpha values for the residency programs’ Now culture types ranged from 0.79 to 0.92 and the Preferred culture types ranged from 0.86 to 0.95.

These results suggest that the dominant residency program Now culture type is the Clan and the least prevailing Now culture type is the Market. For the Preferred culture type, the dominant Preferred culture type is the Clan followed by the Adhocracy, the Hierarchy, and the Market.

### Residents’ perceptions of their programs’ feedback culture (FEEDME-Culture survey)

The Mann-Whitney U tests results denoted that there were no significant differences on the FEEDME-Culture mean score between the Internal Medicine and Pediatrics residency programs (p > 0.05) from different institutions (i.e., MMC vs. BIA internal residents, and MMC vs. BIB pediatric residents).

### Relationship between residency programs’ feedback culture and now culture type

We completed a multiple regression analysis to assess the relationship between the residency programs’ FEEDME-Culture mean scores (as the dependent variable) and Now culture types (Clan, Adhocracy, Market, and Hierarchy). These Now culture types variables were significantly related to the feedback culture mean scores, F (3, 110) = 7.87, p = .000, Adjusted R^2^ = .154. The VIF value was 1.184 that led to a conclusion there was no multicollinearity. The results showed that the Hierarchy Now culture type was significantly related to the feedback culture mean score (B = −.023, p < .01). For every one unit increase in the Hierarchy Now culture type, the FEEDME-Culture mean score decreases by 0.023 units.

### Differences in organizational and feedback cultures between specialties at one institution

We conducted MANOVA analysis with only the Maine Medical Center (n = 86) OCAI and FEEDME-Culture data (one outlier case of the Hierarchy Now culture variable was deleted). We focused on only Maine Medical Center because of the small number of specialties and residents from the two Boston institutions, and the potential for confounding factors between institutions. Findings from the initial correlation analysis indicated that the FEEDME-Culture mean score was minimally and positively correlated with the Clan Now culture type (r = .18) and moderately and negatively correlated with Hierarchy Now culture type (r = −0.30). The rest of the culture type variables were not significantly correlated and therefore, they were excluded from the MANOVA analysis. All assumptions for MANOVA tests were met.

A statistically significant MANOVA effect was obtained, Pillai’s Trace = .43, F (5, 80) = 2.71, p < .001. The multivariate effect size estimate was 0.145. Furthermore, subsequent one-way ANOVA was not significant for the Hierarchy Now culture type [F (5, 80) = 2.1, p = .07] but they were statistically significant for the Clan Now culture type [F (5, 80) = 3.2, (p = .011] and FEEDME-Culture mean score [F (5, 80) = 4.83, (p = .001], with effect sizes (partial η2) of .167 and.232.

**Figure 1.**
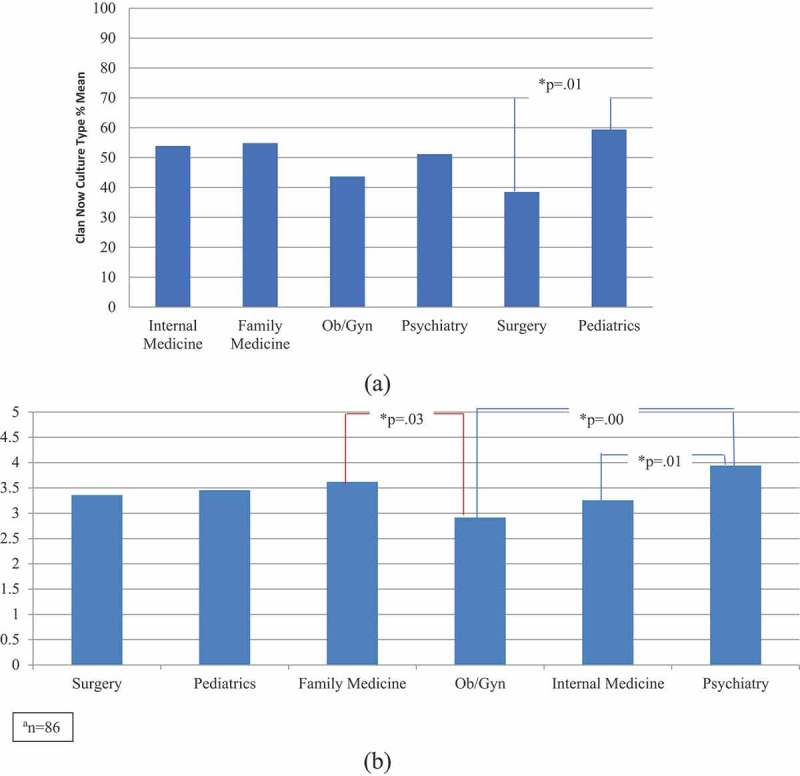
a) Posthoc analysis results: clan now culture type by residency programs at Maine Medical Center^a^. b) Posthoc analysis results: FEEDME-Culture mean score by residency programs at Maine Medical Center^a.^ a = 86.

Finally, we performed a series of posthoc analyses (Tukey) to examine mean difference comparisons across the two significant Clan Now culture type and FEEDME-Culture mean score dependent variables and all six specialties at only Maine Medical Center (n = 86). The results revealed that a few posthoc mean comparisons were statistically significant (Figure 1a and 1b). The Clan culture type was more dominant in the Pediatrics than in the Surgery residency program (p = 0.01). On an average, feedback culture was more established in the Psychiatry than in the OB/GYN (p = 0.01) and Internal Medicine residency (p = 0.01) programs.

## Discussion

The results of our cross-sectional study involving residents from three institutions add to the medical literature by providing insights into the interplay between the residency program culture and the feedback culture, as perceived by residents. The findings suggested that a Clan culture was the current, dominant residency program culture as well as the preferred culture by residents. In addition, residents’ perceptions of a current Hierarchy culture were significantly associated with a negative impact on the feedback culture.

Our first research question (what is the relationship between residents’ perceptions of their program’s organizational culture and feedback culture?) is answered with the correlational results between the OCAI and FEEDME-Culture mean scores. We are not entirely surprised by the findings, as organizations with a strong hierarchical culture are less likely to promote a safe culture where individuals can raise concerns and speak [23]. Residents may perceive a hierarchal culture as a barrier to promoting a healthy feedback exchange. A study using the OCAI survey in 40 U.S. hospitals found that 37.5% had a dominant hierarchal culture, which was negatively associated with attitudes and climate for patient safety [24]. Employees’ perceptions of being at a certain level within an organizational hierarchy may further impact their seeking of informal feedback [25] and the hierarchy may explain employees’ perceptions of the quality of feedback [26]. An organizational culture more similar to the Clan culture, as described in the Competing Values Framework [17] and assessed by the OCAI instrument, has been described as having more frequent informal feedback for employees, higher levels of interaction between superiors and subordinates, and a stronger focus on employee development [27] than a culture more akin to the Hierarchy culture.

Regarding our second research question (are there differences in residents’ perceptions of their programs’ organizational and feedback cultures amongst residents from different disciplines and different institutions?), all residents regardless of discipline and institution perceived and desired a Clan culture, which is more like an extended family, with faculty and residency leadership seen as more mentors and even possibly parental figures. In this type of culture, commitment is high, and loyalty and traditions are valued [17]. Due to the rigorous nature of residency with long hours, residents may desire a Clan culture, which might provide more support and encouragement. Residents were least desirable of a Market culture, which is more results-oriented, and focused on getting the work done. Individuals in Market cultures are competitive and value reputation and success [17]. We also found significant differences in residents’ perceived program culture and feedback culture between specialties at one institution. These findings are consistent with the literature that described the feedback culture as challenging for surgeons [28]. Scholars found that some surgery residents view intimidation and harassment by their surgery teachers as functional educational tools [29].

We believe the generalizability of our findings is strengthened by examining the perceptions of residents from many disciplines and three different institutions. That said, a limitation of our study is that all of the institutions are located in the Northeast and the relatively low overall response rate. In addition, we did not query residents who did not volunteer to participate in the study; these residents might have different perceptions of the residency program or feedback cultures than participating residents. Our findings of an association between only the Clan and Hierarchy cultures and not the other cultures may suggest the OCAI instrument is not refined enough. In addition, our methods do not explain why residents prefer a Clan culture; subsequent qualitative studies may help understand their perspectives. Last, our surveys assess learners’ perceptions only, which may not reflect the actual residency program and feedback culture.

### Conclusion

With the growing emphasis on the need to establish an educational alliance between learners and teachers to promote effective feedback, understanding the culture within which this dialogic interaction occurs becomes important. Our findings indicate that residents across different disciplines and institutions prefer a more ‘family’-type culture, which may promote more beneficial feedback for residents. Hierarchy cultures within residencies appear to be negatively associated with the perception of effective feedback. Addressing cultural barriers to feedback may be vital to improving the effective exchange of feedback in the clinical learning environment. Future studies may explore how residency programs could change from a hierarchal culture to a more clan culture.
